# Pharmacist beliefs about antimicrobial resistance and impacts on antibiotic supply: a multinational survey

**DOI:** 10.1093/jacamr/dlac062

**Published:** 2022-08-24

**Authors:** Amy Hai Yan Chan, Kebede Beyene, Chloe Tuck, Victoria Rutter, Diane Ashiru-Oredope

**Affiliations:** Commonwealth Pharmacists’ Association, London, UK; School of Pharmacy, University of Auckland, Grafton, Auckland, New Zealand; UCL School of Pharmacy, University College London, London, UK; School of Pharmacy, University of Auckland, Grafton, Auckland, New Zealand; Commonwealth Pharmacists’ Association, London, UK; Commonwealth Pharmacists’ Association, London, UK; Commonwealth Pharmacists’ Association, London, UK

## Abstract

**Background:**

Pharmacists have important antimicrobial stewardship (AMS) roles yet limited literature exists on pharmacists’ knowledge and beliefs about antimicrobial resistance (AMR) and antimicrobials and how these beliefs influence antimicrobial supply in different countries.

**Methods:**

A cross-sectional survey was disseminated to pharmacists around the world via the Commonwealth Pharmacists’ Association and related networks. Data were collected on demographics, antibiotic supply practices, and knowledge and beliefs about AMR.

**Results:**

A total of 546 pharmacists responded from 59 countries, most commonly from Africa (41%) followed by Asia (26%) and Oceania (22%). Respondents supplied a mean of 46 ± 81 antibiotic prescriptions/week, 73%±35% of which were given in response to a prescription. Overall, 60.2% dispensed antibiotics at least once without a prescription. Respondents had good knowledge (mean 9.6 ± 1.3 (out of 12), and held positive beliefs about AMR [mean 3.9 ± 0.6 (out of 5)]. Knowledge about antibiotics and beliefs about AMR were positively correlated. The odds of supplying antibiotics without a prescription were 7.4 times higher among respondents from lower income countries [adjusted odds ratio (AOR) = 7.42, 95% CI 4.16–13.24]. Conversely, more positive AMR beliefs were associated with a lower odds of supplying antibiotics without a prescription (AOR = 0.91, 95% CI 0.86–0.95).

**Conclusions:**

Most pharmacists had the good knowledge about antibiotics and positive beliefs about AMR. These beliefs were influenced by knowledge, work setting, and country income. A proportion of respondents provided antibiotics without a prescription; the likelihood of this occurring was higher in those who held more negative beliefs about AMR.

## Introduction

Antimicrobial resistance (AMR) is one of the greatest threats to public health and global security, with significant societal impact across both high- and low-middle-income countries.^1,2^ By 2050, an estimated 10 million people globally will die each year from drug-resistant infections if no action is taken now.^3^ This is particularly concerning as the use of antibiotics in many settings is increasing.^2^ AMR is driven by overuse of antimicrobials, or inappropriate use in situations where antimicrobials are not indicated, in both humans and animals, or where use is not in accordance with guidelines, or deemed clinically unnecessary (such as for self-limiting, viral upper respiratory tract infections).^4,5^

Within the supply chain of antimicrobials, a diverse range of healthcare professionals are usually involved, from the prescribing clinician, to dispensing by pharmacists, to administration by nurses or patients who are self-medicating. Pharmacists have been increasingly recognized as key members of the antimicrobial supply process^6^ with important antimicrobial stewardship (AMS) roles within the multidisciplinary team. Importantly, pharmacists are uniquely positioned as one of the most easily accessible health professionals (particularly in the community), offering key opportunities to provide medication education to patients where other clinical infrastructure is lacking or access to reliable health information is difficult.^7,8^ This is particularly relevant when most of our antibiotic use and prescribing is in primary care and for respiratory tract infections.^9^ Current evidence supports the role of pharmacists in reducing unnecessary prescribing, and increasing the appropriateness of prescribing, and the integration of pharmacist-led AMS practices in the community,^10,11^ for example through pharmacist-led management of uncomplicated infections, has been shown to be effective and well-received by patients.^12^ The World Health Organization (WHO) recognizes the importance of fostering global AMS through multidisciplinary health providers, in order to prevent emergence of further resistance,^4^ with a need for countries to adopt global action plans on AMR that focus on effective AMS by all health providers.^13^

In line with this, many AMR interventions have focused on changing health provider prescribing, such as implementation of prescribing guidelines, supply restrictions on various antimicrobials,^14^ and predominantly health-provider education through materials or meetings.^15^ However, these efforts to tackle AMR have varied in effectiveness,^15^ with education alone often having little or limited effect in terms of generating sustainable long-term improvement in antibiotic prescribing.^16,17^ This is because passive education or information provision alone often does not lead to behaviour change; and even if it does, behaviour change is likely to be short-term ‘unless motivators and values become firmly rooted and norms that support lasting change are established within population’.^18^

This may be because sub-optimal antimicrobial use in many settings is primarily influenced by behavioural drivers,^19^ and simply providing information is not sufficient to change behaviour on a sustainable basis.^20^ To change behaviours such as inappropriate antimicrobial supply and use, interventions need to go beyond information provision to understanding and addressing the psychological and behavioural drivers of antimicrobial use for the health professional.^19^ For example, as antimicrobials become increasingly accessible, they may be overly used by healthcare professionals to meet shortfalls in other healthcare resources and to prevent infection outbreaks where these are of imminent concern.^2,21,22^ In the context of community pharmacy, motivational drivers associated with running a sustainable business may conflict with optimal antimicrobial stewarding behaviour. For example, healthcare providers may be motivated to provide antimicrobials to secure customer loyalty, in the face of increased customer demand, and to turnover stock.^22,23^

Inappropriate antimicrobial supply can also occur when patients request or expect antibiotic treatment for situations where antibiotics are not indicated. Studies show that health providers often prescribe or supply antimicrobials to meet patient expectations and demands, even when they may not be necessary.^24–29^ Coenen *et al.*^30^ found that patient expectations and demands were a top influencing factor in the supply of antimicrobials by clinicians, even when clinicians were aware that the supply was not appropriate. One study found that 42% of physicians stated they would prescribe an antimicrobial when unsure if the infection was viral or bacterial.^31^ Other literature highlights that health professionals and students often have poor knowledge of AMR,^31,32^ with one study reporting that more than half of community pharmacists and physicians had poor knowledge of AMR.^33^ Even though AMR awareness is recognized by most health providers as an important public health issue,^33^ this awareness does not consistently translate into reduced antibiotic prescribing or dispensing, or involvement in AMS initiatives.^14,24^ These behaviours are not well understood and are important to explore as these can influence an individual’s motivation and beliefs about antimicrobials, and antimicrobial use.^34^ Despite the key role that pharmacists play in AMS, limited literature exists on pharmacists’ knowledge and beliefs about AMR and antimicrobials, and how these beliefs are linked with actual antimicrobial supply practices. These antimicrobial supply practices are likely to vary by country due to differences in antimicrobial regulation and cultural practices, yet little has been published on how beliefs and antimicrobial supply vary between countries.

The aim of this study was to explore how pharmacists across the world perceive AMR and antimicrobials, and how these beliefs influence antibiotic supply practices. Specifically, we explored how beliefs about antibiotics and AMR vary between countries, and what factors predicted differences in beliefs and supply practices.

## Materials and methods

A cross-sectional survey was developed for health professionals based on previously validated surveys on antimicrobials and medicines-related behaviours.^35,36^ Respondents were invited to answer an initial set of questions on their antibiotic use behaviour and knowledge based on the WHO ‘Antibiotic resistance: multi-country public awareness survey’,^37^ and adapted from a survey originally developed for physicians by Teixeira Rodrigues *et al*.^36^ These measures are described below.

### Demographics

Respondents were asked to choose: the country from which they were responding; age; gender (male, female, other, prefer not to say); how long they have been working in pharmacy (less than 1 year, 1–4 years, 5–9 years, 10–19 years, 20 or more years), and which setting they are currently working in (hospital, community pharmacy, academia/research, other e.g. private sector or industry). Based on the respondent’s self-reported country, the country’s income classification was allocated using World Bank country income data from 2017 (https://datatopics.worldbank.org/world-development-indicators/the-world-by-income-and-region.html), allowing countries to be classified as high, upper-middle, low-middle or low-income. For each of these countries, prescribing regulations were checked to find out if formal legislation existed to regulate antibiotic supply, and investigate whether this would affect antibiotic supply practices.

### Antibiotic supply practices

Respondents were asked ‘How often do you dispense/prescribe antibiotics per week on average?’ as an absolute numerical value. For those who identified their role as ‘pharmacist’, they were asked to indicate from a scale of 1% to 100%, their response to: ‘Of the antibiotics you supply, roughly what percentage were given in response to you receiving a prescription?’.

### Knowledge

Knowledge about antibiotics was also measured using an antibiotic knowledge questionnaire adapted from the WHO ‘Antibiotic resistance: multi-country public awareness survey’. Respondents were asked to identify whether antibiotics could be used for each of a list of twelve health conditions [HIV/AIDS, gonorrhoea, bladder infection or urinary tract infection (UTI), diarrhoea, cold and flu, fever, malaria, measles, skin or wound infection, sore throat, body aches, headaches] for which higher scores indicate greater knowledge. This questionnaire had a maximum score of 12.

### Beliefs about AMR

Beliefs about antibiotics and of AMR (specifically about antibiotic resistance) were evaluated using an online questionnaire adapted from the WHO survey and other literature.^36,37^ The questionnaire comprised 11 statements to which respondents could indicate their level of agreement via a 5 point scale ranging from ‘agree strongly’ to ‘disagree strongly’, with a neutral mid-point. Higher scores indicate stronger agreement that AMR is important to address. Examples of items include ‘Antibiotic resistance is an important public health problem in our setting’, and ‘The prescription of an antibiotic to a patient does not influence the possible appearance of resistance’ (reverse-scored). The maximum sum score was 55, indicating a strong belief that AMR is an important issue.

### Survey dissemination

The survey was disseminated to pharmacists via the Commonwealth Pharmacists’ Association, and related personal and research networks. The survey was launched during the WHO’s World Antibiotic Awareness Week, a campaign that aims to raise the awareness of the importance of preserving antibiotics by optimizing use and thus reducing AMR.

### Statistical analysis

Data were analysed using SPSS version 27. The data were summarized using descriptive statistics. Groups were formed for gender of the participant (male, female), and participants’ country income (high, upper-middle, low-middle, or low income). Knowledge and beliefs scores were calculated by summing the score of each item. The internal consistency of Likert items assessing beliefs of AMR was checked using Cronbach’s alpha. A multiple linear regression model was used to examine predictors of beliefs about AMR. Predictor variables used in linear regression were sex (male, female), income classification (high/upper-middle income, low/low-middle income), work experience (<5 years, 5–9 years, ≥10 years), work setting (community, hospital, academia/research, other), and knowledge about antibiotics. Regression coefficients with 95% CIs were derived from the linear regression model, representing the average change in beliefs about AMR score per unit change in the respective predictor variable. The adjusted R^2^ value was reported to provide information about the percentage of variance explained by the model. Multivariable logistic regression was used to identify determinants of dispensing of antibiotics without a prescription. The outcome variable was dichotomized using responses to the question ‘Of the antibiotics you supply, roughly what percentage are given in response to you receiving a prescription?’ into ‘never dispensed antibiotics without a prescription’ (coded as ‘0’) or ‘dispensed antibiotics at least once without a prescription’ (coded as ‘1’). Respondents who stated that they did not dispense any antibiotics were excluded from analysis.

In addition to the aforementioned predictor variables, beliefs about AMR score was included as a potential predictor of dispensing antibiotics with a prescription. Logistic regression results were reported as adjusted odds ratios with 95% CIs. Age was highly correlated with work experience; thus, age was excluded from all regression models to avoid multi-collinearity. All tests were two-tailed, and a *P *< 0.05 was considered statistically significant.

### Ethics

According to an online review by the UK NHS Research Ethics Committee, and the University College London ethics policies, no further ethics approval was deemed necessary for this study, as the online survey did not collect any identifying data, and involved the use of non-sensitive, completely anonymous survey procedures.^38^ All respondents provided written consent for collection of data via the survey for research purposes and for anonymous publication of the data.

## Results

A total of 546 pharmacists [age (mean ± SD) 39.2 ± 12.5 years; 51.5% female] responded from 59 countries, with the most common region of respondents being Africa (41%) followed by Asia (26%) and Oceania (22%) (Table 1). Most respondents were from low-middle income countries (44%), practised in countries where prescribing regulations existed for antibiotics (94%), had 10 years or more of work experience (51%), and practised in either hospital or community (66%). Participants reported supplying a mean of 46 ± 81 antibiotic prescriptions per week, of which 73% ± 35% were given in response to a prescription. Overall, 150 respondents (39.8%) never dispensed antibiotics without a prescription, and 227 (60.2%) dispensed antibiotics at least once without a prescription. There were 169 people who responded ‘none’ to the question ‘How often do you dispense/prescribe antibiotics per week on average?’.

**Table 1.** Sociodemographic characteristics and antibiotic-dispensing practices (*N *= 546)

Characteristic	Value^a^
Age, years, mean ± SD	39.3 ± 12.5
Gender	
Male	263 (48.2)
Female	281 (51.5)
Missing	2 (0.4)
Region	
Africa	223 (40.8)
Asia	139 (25.5)
Oceania	120 (22.0)
Europe	38 (7.0)
North America	10 (1.8)
South America	10 (1.8)
Missing	6 (1.1)
Country income ranking	
High-income	168 (30.8)
Upper-middle income	77 (14.1)
Low-middle income	240 (44.0)
Low income	55 (10.1)
Missing	6 (1.1)
Prescribing regulations for antibiotics	
Yes	512 (93.8)
No	34 (6.2)
Work experience	
<5 years	155 (28.4)
5–9 years	97 (17.8)
≥10 years	277 (50.7)
Missing	17 (3.1)
Work setting	
Hospital pharmacy	193 (35.3)
Community pharmacy	166 (30.4)
Academia/Research	53 (9.7)
Other	134 (24.5)
How often do you dispense/prescribe antibiotics per week on average? (*n *= 394)^b^	
Mean ± SD	45.5 ± 81
Median (IQR)	20 (4–60)
Of the antibiotics you supply, roughly what percentage are given in response to you receiving a prescription? (*n *= 377)^b^	
Mean ± SD	72.3%±34.6%
Median (IQR)	92% (50%–100%)

a Results shown are *n* (%) unless otherwise indicated.

b The base unit is number of prescriptions.

Overall, respondents scored well in the knowledge part of the survey about antibiotics, with mean knowledge scores of 9.6 ± 1.3 (out of 12). Of the conditions tested, respondents most commonly (>90%) recognized the correct treatment for headaches, body aches, measles, HIV/AIDs, cold and flu, skin/wound infections, and bladder infections; respondents most commonly answered incorrectly for malaria, sore throats and diarrhoea (Table 2).

**Table 2.** Which of these conditions do you think can be treated with antibiotics? (*N *= 414)

Condition	Correct answer	Percentage of participants who provided the correct response
Bladder infection or urinary tract infection	True	94.7%
Skin or wound infection	True	92.0%
Gonorrhoea	True	86.2%
Malaria	True	23.7%
Cold and flu	False	91.8%
Sore throats	False	47.1%
HIV/AIDs	False	91.1%
Diarrhoea	False	51.9%
Fever	False	85.7%
Measles	False	94.4%
Body aches	False	98.8%
Headaches	False	99.3%

Mean beliefs about AMR scores for this cohort were 3.9 ± 0.6 (out of 5), indicating that on average, most respondents recognized AMR as important and had good knowledge about factors leading to AMR. The overwhelming majority of the participants (92%) strongly agreed with the statement ‘Antibiotic resistance is an important public health problem in our setting’, and 81% of participants strongly agreed with the statement ‘Dispensing antibiotics without a prescription should be more closely controlled’. A detailed description of the Likert items assessing beliefs about AMR and their responses is provided in Table S1 (available as Supplementary data at *JAC-AMR* Online). The internal consistency of all items forming beliefs about AMR score was satisfactory, with Cronbach’s alpha values of 0.7.

Table 3 presents the results of the multivariable linear regression model, which explored factors associated with beliefs about AMR. The independent variables significantly predict beliefs about AMR [F(8, 400) = 18.9, *P *< 0.001], showing that the regression model was a good fit of the data. The model explained 26% of the variability in beliefs about AMR scores (adjusted R^2 ^= 0.260). There was a positive correlation between knowledge about antibiotics and beliefs about AMR, meaning that the better knowledge respondents had, the fewer misperceptions they had relating to AMR. Beliefs about AMR scores increased by 1.11 points (95% CI 0.657–1.558) for every unit increase in antibiotic knowledge score. Compared with community pharmacists, hospital pharmacists held more positive beliefs about AMR (β = 2.864, 95% CI = 1.573–4.154). Conversely, respondents from low and low-middle income countries had lower scores for the beliefs about the AMR domain compared with respondents from upper-middle and high income countries (β = −4.059, 95% CI −5.289 to −2.829) (Table 3). There was no relationship between work experience and beliefs about AMR.

**Table 3.** Multivariable linear regression examining predictors of beliefs about AMR (*N *= 409)

Characteristic	B	Beta	*P* value	95% CI	
				Lower	Upper
Female	0.299	0.023	0.622	−0.892	1.490
Country Income Rank (Ref = High Income Country)					
Low-income country	−4.059	−0.312	**<0**.**001**	**−5**.**289**	**−2**.**829**
Work experience (Ref <5 years)					
5–9 years	1.148	0.069	0.168	−0.486	2.782
>9 years	0.893	0.069	0.178	−0.407	2.192
Knowledge about antibiotics	1.107	0.220	**<0**.**001**	**0**.**657**	**1**.**558**
Work setting (Ref = Community Pharmacy)					
Hospital Pharmacy	2.864	0.214	**<0**.**001**	**1**.**573**	**4**.**154**
Academia/Research	−0.736	−0.030	0.509	−2.927	1.455
Other	1.178	0.071	0.140	−0.388	2.745

*P* values <0.05 are shown in bold.

Table 4 shows the results of the multivariable logistic regression model examining factors associated with the supply of antibiotics without a prescription. The model explained enough variation in beliefs about AMR to be considered as a useful model (χ^2^ = 132.73, df = 8, *P *< 0.001), with Nagelkerke R^2 ^= 0.406 (i.e. the model explained 40% of the variation in beliefs). In terms of country income, the odds of supplying antibiotics without a prescription were 7.4 times higher among respondents from lower income countries compared with their counterparts from higher income countries [adjusted odds ratio (AOR) = 7.418, 95% CI 4.156–13.239]. Conversely, higher scores on the beliefs about AMR scale (i.e. more-positive beliefs) were associated with a lower odds of supplying antibiotics without a prescription (AOR = 0.906, 95% CI 0.861–0.953). However, antibiotic knowledge score was not associated with antibiotic supply without a prescription, nor was years of work experience or work setting.

**Table 4.** Multivariable logistic regression examining predictors of dispensing antibiotics without a prescription (*N *= 372)

Characteristic	Adjusted odds ratio (95% CI)	*P* value
Female sex	1.110 (0.631–1.952)	0.718
Country Income Rank (Ref = High income country)		
Low-income country	**7.418** (**4.156–13.239)**	**<0**.**001**
Work experience (Ref <5 years)		0.616
5–9 years	0.815 (0.378–1.756)	0.601
>9 years	0.740 (0.406–1.348)	0.325
Work setting (Ref = Community Pharmacy)		0.129
Hospital Pharmacy	0.812 (0.455–1.450)	0.482
Other	1.691 (0.821–3.485)	0.154
Knowledge about antibiotics score	0.972 (0.780–1.211)	0.800
Beliefs about AMR score	**0.906** (**0.861–0.953)**	**<0**.**001**

Results where the *P* value is <0.05 are shown in bold.

## Discussion

This is one of the first studies to examine pharmacist beliefs about AMR, with respondents from 59 different countries, and how this influences actual antibiotic supply practices. Previous literature have highlighted the importance of considering an individual’s beliefs and perceptions about antibiotics and AMR, as this can influence their behaviour, for example, individuals with misconceptions about antibiotics had substantially increased odds of antibiotic misuse such as self-medication with antibiotics and obtaining antibiotics without prescription.^39–42^ Similarly, there have been several studies that have explored pharmacist knowledge and beliefs about antibiotics and AMR;^43,44^ however, few examined how pharmacist decisions relating to antibiotic supply were influenced by their personal beliefs about antibiotics and AMR. A study in Thailand conducted over 10 years ago found that a pharmacists’ intention to dispense antibiotics for upper respiratory infections was strongly influenced by attitude.^45^ However, in Thailand at the time of the study completion, community pharmacists were allowed to dispense antibiotics without prescription, which is not the current case in most countries, where antibiotic supply is regulated by law. As such, whether beliefs also show the same association with antibiotic supply practices in other countries now is uncertain. More recent qualitative studies with pharmacists suggest that there are certain attitudes that could lead to antibiotic dispensing without a prescription.^46,47^ A Portuguese study with 32 pharmacists identified attitudes such as complacency, precaution and external responsibility as being associated with supply of antibiotics without a prescription, for example, supplying antibiotics for certain infections where pharmacists felt they knew doctors would prescribe.^46^ Similarly, a study with 17 pharmacists in Mozambique identified reasons for non-prescribed antibiotic dispensing as being linked to pharmacists beliefs that patients expect antibiotics, or about physicians’ prescribing practices.^47^

Similar to these qualitative studies, we found a significant negative association between beliefs about AMR and the supply of antibiotics without a prescription, meaning those with more positive and accurate beliefs about AMR were less likely to supply antibiotics without a prescription. This association was significantly influenced by the country in which the pharmacist was practising; the odds of this occurring were over 7 times higher in pharmacists practising in low/low-middle income countries. This finding has important implications when considering the development of global health policies relating to AMR as this could have implications for public access to necessary antimicrobials even though stricter regulation could reduce unnecessary antimicrobial use. AMS initiatives should consider a pharmacist’s individual beliefs about AMR but also the country context in which pharmacists practise. Key potentially modifiable factors that can influence and modify pharmacist beliefs about AMR were identified from our study, including knowledge and work setting. Our study found that knowledge about antibiotics significantly predicted beliefs about AMR, and that hospital pharmacists held more accurate beliefs about how AMR arises and more positive beliefs about the importance of addressing AMR, potentially because hospital pharmacists have more opportunities to be exposed to in-house continuing education programmes and/or AMS campaigns, and also less financial pressure to increase sales to sustain a business. This suggests that interventions that equip pharmacists with better knowledge can potentially shape their beliefs and influence their antibiotic supply practices. This is in line with the findings from a recent 2018 scoping review of AMS initiatives in community pharmacies.^48^ The review found that supply of antibiotics without a prescription was commonplace in community pharmacies, primarily driven by pharmacist perceptions about their role to support customers to obtain medical services that they need. The authors suggested that educational interventions directed towards improved patient–pharmacist interactive sessions are needed for exercising appropriate and rational use of antibiotics.

Our findings support those reported in previous international studies and add to the literature by providing data from 59 different countries. Similar to findings from a large study conducted across Europe, we found that knowledge of antibiotics and AMR varied widely.^49^ One of the key strengths of this study was having data from several different global regions, namely Africa, Asia, and Oceania, allowing exploration of pharmacist perceptions and antibiotic practices in different settings. Our study also has a large sample size of over 500 respondents, though not all pharmacists were active suppliers of antibiotics. Additionally, whilst we found significant associations between knowledge and beliefs, there were some questions in the knowledge questionnaire that were ambiguous in terms of whether antibiotics can be used to treat the condition. For example, the low-scoring conditions of diarrhoea, malaria and sore throat may reflect the fact that antibiotics can be used to treat these conditions depending on the aetiology, even though it may not be the first-line or most common treatment of choice. Our choice to use this knowledge quiz was based on the original WHO survey, however, future studies may benefit from using a validated questionnaire or alternative method of assessing antibiotic and AMR knowledge. Additionally, our sample was limited to only respondents who were able to complete the survey in English, which may explain the low respondent numbers from Europe and South America. The survey could have benefited from being translated into other languages such as Spanish, Mandarin, Arabic, French, etc. to increase responses from countries where English is not the primary language.

Overall, this study found that most pharmacists had good knowledge about antibiotics and positive beliefs about AMR, with the majority recognizing the correct situations where antibiotics would be warranted for treatment and that AMR is an important public health issue that is influenced by the inappropriate prescription of antibiotics. These beliefs were influenced by respondent’s knowledge about antibiotics, work setting, and country income. Importantly, a proportion of respondents still provided antibiotics without a prescription; the likelihood of this occurring was increased in those who scored lower on the questionnaires about beliefs about AMR. Initiatives to improve antibiotic supply by pharmacists should aim to address misplaced beliefs about AMR and improve knowledge. Strategies should be tailored to the individual’s personal beliefs and different approaches are likely needed in lower and middle-income countries compared with higher income settings.

## Supplementary Material

dlac062_Supplementary_Data

## Transparency declarations

A.H.Y.C. reports grants and personal fees from Janssen-Cilag, consultancy fees from Spoonful of Sugar Ltd, grants from A+ charitable trust (Auckland District Health Board), Maurice and Phyllis Paykel trust, Universitas 21, New Zealand Pharmacy Education Research Fund, Auckland Academic Health Alliance, Asthma UK, the University of Auckland, Health Research Council, and Oakley Mental Health Foundation, outside the submitted work. A.H.Y.C. is also the recipient of the Robert Irwin Postdoctoral Fellowship and the Senior Research Fellowship from the Auckland Medical Research Foundation. The remaining authors have none to declare.

## Supplementary data

Appendix A is available as Supplementary data at *JAC-AMR* Online.
