# Supplementary material for: Pharmacist beliefs about antimicrobial resistance and impacts on antibiotic supply: a multinational survey
Source: JAC Antimicrob Resist. 2022 Aug 24;4(4):dlac062. doi: 10.1093/jacamr/dlac062 (PMC9400174; doi:10.1093/jacamr/dlac062)
Supplement: dlac062_Supplementary_Data [file dlac062_supplementary_data.docx]

**Supplementary data**

**Appendix A**

**World Antibiotics Awareness Week Survey**

**Country** (drop down)

**Age** (limit to whole numbers)

**Gender**

- Male
- Female
- Other
- Prefer not to say

**How long you have been working in pharmacy?**

- Less than 1 year
- 1 - 4 years
- 5 - 9 years
- 10 - 19 years
- 20 or more years

**In which setting are you currently working in?**

- Hospital
- Community pharmacy
- Academia / Research
- Private sector
- Industry
- Other

**If other, please specify below.**

Free text

**Which of these conditions do you think can be treated with antibiotics?**

[Tick all that apply]

1. HIV/AIDS
2. Gonorrhoea
3. Bladder infection or urinary tract infection (UTI)
4. Diarrhoea
5. Cold and flu
6. Fever
7. Malaria
8. Measles
9. Skin or wound infection
10. Sore throat
11. Body aches
12. Headaches
13. **How often do you dispense/prescribe antibiotics per week on average?**

…………./week [number only]

**Of the antibiotics you supply, roughly what percentage are given in response to you receiving a prescription?**

Scale 1-100%

1. **Which of these conditions can be treated with antibiotics?**  (select all that apply)
2. HIV/AIDS
3. Gonorrhoea
4. Bladder infection or urinary tract infection (UTI)
5. Diarrhoea
6. Cold and flu
7. Fever
8. Malaria
9. Measles
10. Skin or wound infection
11. Sore throat
12. Body aches
13. Headaches
14. **Below are some statements about antibiotics. Please indicate below whether you agree with these. There are no right or wrong answers, we are simply interested in your views.**

High scores, negative AMR beliefs

|  |  | **Agree Strongly** | **Agree**  **Slightly** | **Neither agree nor disagree** | **Disagree Slightly** | **Disagree Strongly** |
| --- | --- | --- | --- | --- | --- | --- |
| HCP1 | Antibiotic resistance is an important Public Health problem in our setting | 5 | 4 | 3 | 2 | 1 |
| HCP2 | The prescription of an antibiotic to a patient does not influence the possible appearance of resistance | 5 | 4 | 3 | 2 | 1 |
| HCP3 | I believe new antibiotics will be developed to solve the problem of resistance | 5 | 4 | 3 | 2 | 1 |
| HCP4 | In case of doubt, it is preferable to use a wide-spectrum antibiotic to ensure that the patient is cured of an infection | 5 | 4 | 3 | 2 | 1 |
| HCP5 | In situations of doubt as to whether a disease might be of bacterial aetiology, it is preferable to provide an antibiotic | 5 | 4 | 3 | 2 |  |
| HCP6 | I frequently provide antibiotics because patients insist on it | 5 | 4 | 3 | 2 | 1 |
| HCP7 | I sometimes provide antibiotics so that patients continue to trust me | 5 | 4 | 3 | 2 | 1 |
| HCP8 | If a patient feels that he/she needs antibiotics, he/she will manage to obtain them at another pharmacy if I do not provide | 5 | 4 | 3 | 2 | 1 |
| HCP9 | Two of the main causes of the appearance of antibiotic resistance are patient self-medication and antibiotic misuse; our profession cannot prevent this | 5 | 4 | 3 | 2 | 1 |
| HCP10 | Dispensing antibiotics without a prescription should be more closely controlled | 5 | 4 | 3 | 2 | 1 |
| HCP11 | The phenomenon of resistance to antibiotics is mainly a problem in hospital not community settings | 5 | 4 | 3 | 2 |  |

**Table S1. Percentage distribution of participants' scores on items assessing beliefs about AMR**

| **Likert Item** | Disagree Strongly | Disagree Slightly | Neither agree nor disagree | Agree Slightly | Agree Strongly |
| --- | --- | --- | --- | --- | --- |
| Antibiotic resistance is an important Public Health problem in our setting | 0.0% | 0.2% | 0.2% | 7.5% | 92.0% |
| The prescription of an antibiotic to a patient does not influence the possible appearance of resistance | 51.9% | 18.1% | 8.9% | 9.9% | 11.1% |
| I believe new antibiotics will be developed to solve the problem of resistance | 16.4% | 22.5% | 21.7% | 27.1% | 12.3% |
| In case of doubt, it is preferable to use a wide-spectrum antibiotic to ensure that the patient is cured of an infection | 25.1% | 19.8% | 14.5% | 25.4% | 15.2% |
| In situations of doubt as to whether a disease might be of bacterial aetiology, it is preferable to provide an antibiotic | 41.5% | 26.3% | 13.0% | 15.2% | 3.9% |
| I frequently provide antibiotics because patients insist on it | 63.5% | 14.7% | 10.6% | 6.8% | 4.3% |
| I sometimes provide antibiotics so that patients continue to trust me | 72.5% | 11.1% | 10.4% | 2.9% | 3.1% |
| If a patient feels that he/she needs antibiotics, he/she will manage to obtain them at another pharmacy if I do not provide | 20.8% | 10.9% | 16.9% | 24.2% | 27.3% |
| Two of the main causes of the appearance of antibiotic resistance are patient self-medication and antibiotic misuse; our profession cannot prevent this | 38.2% | 23.4% | 4.1% | 16.7% | 17.6% |
| Dispensing antibiotics without a prescription should be more closely controlled | 1.4% | 1.2% | 5.3% | 11.6% | 80.4% |
| The phenomenon of resistance to antibiotics is mainly a problem in hospital not community settings | 68.4% | 17.6% | 4.8% | 5.6% | 3.6% |

Responses for each item were presented on a 5-point Likert scale ranging from 1 (Disagree Strongly) to 5 (Agree Strongly).
